# Oxidative Stress and Antioxidant Strategies: Relationships and Cellular Pathways for Human Health

**DOI:** 10.3390/cells13221871

**Published:** 2024-11-11

**Authors:** Alessia Remigante, Rossana Morabito

**Affiliations:** Department of Chemical, Biological, Pharmaceutical and Environmental Sciences, University of Messina, 98166 Messina, Italy; rmorabito@unime.it

Chronic diseases and aging have increased significantly in recent decades. These pathological states are produced by different causes, and a common factor in most of them is oxidative stress. Oxidative stress is defined as an imbalance between the oxidative state, mainly due to the formation of reactive species (RS), and antioxidant defense mechanisms. However, when excess oxidants are produced, or when the antioxidant defenses that regulate them are ineffective, this balance can be disturbed, resulting in oxidative conditions. Oxidative products are highly reactive and can directly or indirectly modulate the functions of many enzymes and transcription factors through a complex signaling cascade. This phenomenon increases with age and affects the normal functioning of numerous cells and tissues. Due to the broad and profound biological effects of RS, numerous experimental and clinical studies have focused their attention on the involvement of oxidative stress as a key regulator in the chronic disease state and aging.

This Special Issue will investigate the molecular mechanisms underlying oxidative stress, as well as pathophysiological consequences in cell and tissue function, in order to open new avenues in therapy and drug design (natural or synthetic). Here, we offer an overview of the content of this Special Issue, which contains two reviews and seven original articles:

1. Chlorine (Cl_2_) exposure poses a significant risk to ocular health, with the cornea being particularly susceptible to its corrosive effects. Antioxidants, known for their ability to neutralize reactive oxygen species (ROS) and alleviate oxidative stress, were explored as potential therapeutic agents to counteract chlorine-induced damage. In vitro experiments using human corneal epithelial cells showed decreased cell viability due to chlorine-induced ROS production, which was reversed by antioxidant incubation. The mitochondrial membrane potential decreased due to both low and high doses of Cl_2_ exposure; however, it was recovered through antioxidants. The wound scratch assay showed that antioxidants mitigated impaired wound healing after Cl_2_ exposure. In vivo and ex vivo, after Cl_2_ exposure, increased corneal fluorescein staining indicates damaged corneal epithelial and stromal layers of mice corneas. Likewise, Cl_2_ exposure in human ex vivo corneas led to corneal injury characterized by epithelial fluorescein staining and epithelial erosion. However, antioxidants protected against Cl_2_-induced damage. These results highlight the effects of Cl_2_ on corneal cells using in vitro, ex vivo, and in vivo models while also underscoring the potential of antioxidants, such as vitamin A, vitamin C, resveratrol, and melatonin, as protective agents against acute chlorine toxicity-induced corneal injury. Further investigation is needed to confirm the antioxidants’ capacity to alleviate oxidative stress and enhance the corneal healing process [1].

2. Moderate levels of reactive oxygen species (ROS), such as hydrogen peroxide (H_2_O_2_), fuel tumor metastasis and invasion in a variety of cancer types. Conversely, excessive ROS levels can impair tumor growth and metastasis by triggering cancer cell death. In order to cope with the oxidative stress imposed by the tumor microenvironment, malignant cells exploit a sophisticated network of antioxidant defense mechanisms. Targeting the antioxidant capacity of cancer cells and enhancing their sensitivity to ROS-dependent cell death represent promising strategies for alternative anticancer treatments. Transient Receptor Potential Ankyrin 1 (TRPA1) is a redox-sensitive non-selective cation channel that mediates extracellular Ca^2+^ entry upon an increase in intracellular ROS levels. The ensuing increase in intracellular Ca^2+^ concentration can in turn engage a non-canonical antioxidant defense program or induce mitochondrial Ca^2+^ dysfunction and apoptotic cell death, depending on the cancer type. Herein, the authors describe the opposing effects of ROS-dependent TRPA1 activation on cancer cell fate and propose the pharmacological manipulation of TRPA1 as an alternative therapeutic strategy to enhance cancer cell sensitivity to oxidative stress [2].

3. Nitric oxide (NO) represents a crucial mediator for regulating cerebral blood flow (CBF) in human brain both under basal conditions and in response to somatosensory stimulation. An increase in intracellular Ca^2+^ concentrations ([Ca^2+^]i) stimulates the endothelial NO synthase to produce NO in human cerebrovascular endothelial cells. Therefore, targeting the endothelial ion channel machinery could represent a promising strategy to rescue endothelial NO signaling in traumatic brain injury and neurodegenerative disorders. Allyl isothiocyanate (AITC), a major active constituent of cruciferous vegetables, was found to increase CBF in non-human preclinical models, but it is still unknown whether it stimulates NO release in human brain capillary endothelial cells. In the present investigation, the authors showed that AITC evoked a Ca^2+^-dependent NO release in the human cerebrovascular endothelial cell line, hCMEC/D3. The Ca^2+^ response to AITC was shaped by both intra- and extracellular Ca^2+^ sources, although it was insensitive to the pharmacological blockade of transient receptor potential ankyrin 1, which is believed to be among the main molecular targets of AITC. In accord, AITC failed to induce transmembrane currents or elicit membrane hyperpolarization, although NS309, a selective opener of small- and intermediate-conductance Ca^2+^-activated K+ channels, induced significant membrane hyperpolarization. The AITC-evoked Ca^2+^ signal was triggered by the production of cytosolic, but not mitochondrial, reactive oxygen species (ROS) and supported by store-operated Ca^2+^ entry (SOCE). Conversely, the Ca^2+^ response to AITC did not require Ca^2+^ mobilization from the endoplasmic reticulum, lysosomes, or mitochondria. However, pharmacological manipulation revealed that AITC-dependent ROS generation inhibited plasma membrane Ca^2+^-ATPase (PMCA) activity, thereby attenuating Ca^2+^ removal across the plasma membrane and resulting in a sustained increase in [Ca^2+^]i. Moreover, the AITC-evoked NO release was driven by ROS generation and required ROS-dependent inhibition of PMCA activity. These data suggest that AITC could be exploited to restore NO signaling and restore CBF in brain disorders that feature neurovascular dysfunction [3].

4. Cardiac lipo-toxicity is an important contributor to cardiovascular complications during obesity. Given the fundamental role of the endoplasmic reticulum (ER)-resident Selenoprotein T (SELENOT) for cardiomyocyte differentiation and protection and for the regulation of glucose metabolism, the authors took advantage of a small peptide (PSELT) derived from the SELENOT redox-active motif to uncover the mechanisms through which PSELT could protect cardiomyocytes against lipo-toxicity. To this end, the authors modeled cardiac lipo-toxicity by exposing H9c2 cardiomyocytes to palmitate (PA). The results showed that PSELT counteracted PA-induced cell death, lactate dehydrogenase release, and the accumulation of intracellular lipid droplets, while an inert form of the peptide (I-PSELT) lacking selenocysteine was not active against PA-induced cardiomyocyte death. Mechanistically, PSELT counteracted PA-induced cytosolic and mitochondrial oxidative stress and rescued SELENOT expression that had been downregulated by PA through FAT/CD36 (cluster of differentiation 36/fatty acid translocase), the main transporter of fatty acids in the heart. Immunofluorescence analysis indicated that PSELT also relieved the PA-dependent increase in CD36 expression, while in SELENOT-deficient cardiomyocytes, PA exacerbated cell death, which was not mitigated by exogenous PSELT. On the other hand, PSELT improved mitochondrial respiration during PA treatment and regulated mitochondrial biogenesis and dynamics, preventing the PA-provoked decrease in PGC1-α and increases in DRP-1 and OPA-1. These findings were corroborated by transmission electron microscopy (TEM), revealing that PSELT improved the cardiomyocyte and mitochondrial ultra-structures and restored the ER network. Spectroscopic characterization indicated that PSELT significantly attenuated infrared spectral-related macromolecular changes (i.e., content of lipids, proteins, nucleic acids, and carbohydrates) and also prevented the decrease in membrane fluidity induced by PA. The findings further delineate the biological significance of SELENOT in cardiomyocytes and indicate the potential of its mimetic PSELT as a protective agent for counteracting cardiac lipo-toxicity [4].

5. Mercury is a toxic heavy metal widely dispersed in the natural environment. Mercury exposure induces an increase in oxidative stress in red blood cells (RBCs) through the production of reactive species and alteration of the endogenous antioxidant defense system. Recently, among various natural antioxidants, the polyphenols from extra-virgin olive oil (EVOO), an important element of the Mediterranean diet, have generated growing interest. Here, the authors examined the potential protective effects of hydroxytyrosol (HT) and/or homovanillyl alcohol (HVA) on an oxidative stress model represented by human RBCs treated with HgCl_2_ (10 µM, 4 h of incubation). Morphological changes, as well as markers of oxidative stress, including thiobarbituric acid reactive substance (TBARS) levels, the oxidation of protein sulfhydryl (-SH) groups, methemoglobin formation (% MetHb), apoptotic cells, a reduced glutathione/oxidized glutathione ratio, Band 3 protein (B3p) content, and anion exchange capability through B3p, were analyzed in RBCs treated with HgCl_2_ with or without 10 μM of HT and/or HVA pre-treatment for 15 min. These data show that 10 µM HT and/or HVA pre-incubation impaired both acanthocyte formation, due to 10 µM HgCl_2_, and mercury-induced oxidative stress injury, as well as restored the endogenous antioxidant system. Interestingly, HgCl_2_ treatment was associated with a decrease in the rate constant for SO_4_^2−^ uptake through B3p, as well as MetHb formation. Both alterations were attenuated by pre-treatment with HT and/or HVA. These findings provide mechanistic insights into benefits derived from the use of naturally occurring polyphenols against oxidative stress induced by HgCl_2_ on RBCs. Thus, dietary supplementation with polyphenols might be useful in populations exposed to HgCl_2_ poisoning [5].

6. Aging is a process characterized by a general decline in physiological functions. The high bioavailability of reactive oxygen species (ROS) plays an important role in the aging rate. Due to the close relationship between aging and oxidative stress (OS), functional foods rich in flavonoids are excellent candidates to counteract age-related changes. This study aimed to verify the protective role of Açaì extract in a d-Galactose (d-Gal)-induced model of aging in human erythrocytes. Markers of OS, including ROS production, thiobarbituric acid reactive substance (TBARS) levels, the oxidation of protein sulfhydryl groups, and the anion exchange capability through Band 3 protein (B3p) and glycated haemoglobin (A1c), were analyzed in erythrocytes treated with d-Gal for 24 h, with or without pre-incubation for 1 h with 0.5–10 µg/mL Açaì extract. The results show that the extract avoided the formation of acanthocytes and leptocytes observed after exposure to 50–100 mM d-Gal, respectively, prevented d-Gal-induced OS damage, and restored alterations in the distribution of B3p and CD47 proteins. Interestingly, d-Gal exposure was associated with an acceleration of the rate constant of SO_4_^2−^ uptake through B3p, as well as A1c formation. Both alterations have been attenuated by pre-treatment with the Açaì extract. These findings contribute to clarifying the aging mechanisms in human erythrocytes and propose functional foods rich in flavonoids as natural antioxidants for the treatment and prevention of OS-related disease conditions [6].

7. The second-most common cause of dementia is vascular dementia (VaD). The majority of VaD patients experience cognitive impairment, which is brought on by oxidative stress and changes in autophagic function, which ultimately result in neuronal impairment and death. In this study, the authors examined a novel method for reversing VaD-induced changes brought on by açai berry supplementation in a VaD mouse model. The purpose of this study was to examine the impact of açai berries on the molecular mechanisms underlying VaD in a mouse model of the disease that was created by repeated ischemia–reperfusion (IR) of the whole bilateral carotid artery. Here, the authors found that açai berry was able to reduce VaD-induced behavioral alteration, as well as hippocampal death, in CA1 and CA3 regions. These effects are probably due to the modulation of nuclear factor erythroid 2-related factor 2 (Nrf-2) and Beclin-1, suggesting possible crosstalk between these molecular pathways. In conclusion, the protective effects of açai berry could be a good supplementation in the future for the management of vascular dementia [7].

8. The Nrf2 gene encodes a transcription factor best known for regulating the expression of antioxidant and detoxification genes. A long list of small molecules has been reported to induce Nrf2 protein via Keap1 oxidation or alkylation. Many of these Nrf2 inducers exhibit off-target or toxic effects due to their nature as electrophiles. In searching for non-toxic Nrf2 inducers, the authors found that a culture medium change to fresh DMEM is capable of inducing Nrf2 protein in HeLa, HEK293, AC16, and MCF7 cells. Testing the components of DMEM led to the discovery of L-Cystine as an effective Nrf2 inducer. L-Cystine induces a dose-dependent increase in Nrf2 protein, ranging from 0.1 to 1.6 mM. RNA-seq analyses and RT-PCR revealed the induction of multiple Nrf2 downstream genes, including NQO1, HMOX1, GCLC, GCLM, SRXN1, TXNRD1, AKR1C, and OSGIN1, by 0.8 mM of L-Cystine. The induction of Nrf2 protein was dependent on L-Cystine entering cells via the cystine/glutamate antiporter and the presence of Keap1. The half-life of Nrf2 protein increased from 19.4 min to 30.9 min with 0.8 mM L-Cystine treatment. L-Cystine was capable of eliciting cyto-protection by reducing ROS generation and protecting against oxidant- or doxorubicin-induced apoptosis. As an amino acid derivative, L-Cystine is considered a non-toxic Nrf2 inducer that exhibits the potential for protection against oxidative stress and tissue injury [8].

9. Either extracts, cell-free suspensions, or bacterial suspensions are used to study bacterial lipid peroxidation processes. Along with gas chromatography–mass spectrometry, liquid chromatography–mass spectrometry, and several other strategies, the thiobarbituric acid test is used for the determination of malondialdehyde (MDA) as the basis for the commercial test kits and the colorimetric detection of lipid peroxidation. The aim of the study was to evaluate lipid peroxidation processes levels in the suspensions, extracts, and culture supernatants of Escherichia coli and Salmonella Derby strains. The dependence of the formation of thiobarbituric acid-reactive substance levels in the cell extracts, suspensions, and cell-free supernatants on bacterial species, as well as their concentrations and growth phases, were revealed. The effect of bacterial concentrations on MDA formation was also found to be more pronounced in bacterial suspensions than in extracts, probably due to the dynamics of MDA release into the intercellular space. This study highlights the possible importance of MDA determination in both cell-free suspensions and extracts, as well as in bacterial suspensions, to elucidate the role of lipid peroxidation processes in bacterial physiology, bacteria–host interactions, and host physiology [9].
